# Phenotypic Characterization of Chicken Thymic Stromal Elements

**DOI:** 10.1155/1992/36905

**Published:** 1992

**Authors:** Richard L. Boyd, Trevor J. Wilson, Andrew G. Bean, Harry A. Ward, M. Eric Gershwin

**Affiliations:** 1 Department of Pathology and Immunology Monash University Medical School Commercial Rd. Prahran Victoria 3181 Australia; 2 Department of Internal Medicine/Rheumatology School of Medicine TB 192 University of California Davis California 95616 USA

**Keywords:** Thymus, chicken, monoclonal antibodies, epithelium, stroma

## Abstract

Phenotypic profiles of the thymic stromal components provide an excellent approach to elucidating the nature of the microenvironment of this organ. To address this issue in chickens, we have produced an extensive panel of 18 mAb to the thymic stroma. These mAb have been extensively characterized with respect to their phenotypic specificities and reveal that the stromal cells are equally as complex as the T cells whose maturation they direct. They further demonstrate that, in comparison to the mammalian thymus, there is a remarkable degree of conservation in thymic architecture between phylogenetically diverse species. Eleven mAb reacted with thymic epithelial cells: MUI-73 was panepithelium, MUI-54 stained all cortical and medullary epithelium but only a minority of the subcapsule, MUI-52 was specific for isolated stellate cortical epithelial cells, MUI-62, -69, and -71 were specific for the medulla (including Hassall’s corpusclelike structures), MUI-51, -53, -70, and -75 reacted only with the type-I epithelium, or discrete regions therein, lining the subcapsular and perivascular regions and MUI-58 demonstrated the antigenic similarity between the subcapsule and the medulla. Seven other mAb identified distinct isolated stromal cells throughout the cortex and medulla. Large thymocyte-rich regions, which often spanned from the outer cortex to medulla, lacked epithelial cells. These mAb should prove invaluable for determining the functional significance of thymic stromal-cell subsets to thymopoiesis.


*Developmental Immunology*, 1992, Vol. 2, pp. 51–66
Reprints available directly from the publisher
Photocopying permitted by license only

© 1992 Harwood Academic Publishers GmbH
Printed in the United Kingdom

### Phenotypic Characterization of Chicken Thymic Stromal Elements

RICHARD L. BOYD,*† TREVOR J. WILSON,†‡ ANDREW G. BEAN†, HARRY A. WARD,†
and M. ERIC GERSHWIN‡

*†Department of Pathology and Immunology, Monash University Medical School, Commercial Rd., Prahran, 3181, Victoria, Australia*
*‡Department of Internal Medicine/Rheumatology, School of Medicine TB 192, University of California, Davis, California 95616*

Phenotypic profiles of the thymic stromal components provide an excellent approach to elucidating the nature of the microenvironment of this organ. To address this issue in chickens, we have produced an extensive panel of 18 mAb to the thymic stroma. These mAb have been extensively characterized with respect to their phenotypic specificities and reveal that the stromal cells are equally as complex as the T cells whose maturation they direct. They further demonstrate that, in comparison to the mammalian thymus, there is a remarkable degree of conservation in thymic architecture between phylogenetically diverse species. Eleven mAb reacted with thymic epithelial cells: MUI-73 was panepithelium, MUI-54 stained all cortical and medullary epithelium but only a minority of the subcapsule, MUI-52 was specific for isolated stellate cortical epithelial cells, MUI-62, -69, and -71 were specific for the medulla (including Hassall's corpuscle-like structures), MUI-51, -53, -70, and -75 reacted only with the type-I epithelium, or discrete regions therein, lining the subcapsular and perivascular regions and MUI-58 demonstrated the antigenic similarity between the subcapsule and the medulla. Seven other mAb identified distinct isolated stromal cells throughout the cortex and medulla. Large thymocyte-rich regions, which often spanned from the outer cortex to medulla, lacked epithelial cells. These mAb should prove invaluable for determining the functional significance of thymic stromal-cell subsets to thymopoiesis.

KEYWORDS: Thymus, chicken, monoclonal antibodies, epithelium, stroma.

### INTRODUCTION

The generation of a functionally competent, complete repertoire of MHC-educated T lymphocytes is dependent on an intact thymus. This intrathymic differentiation of bloodborne precursors involves a complex series of interactions between the maturing thymocytes and subsets of the thymic stromal cells (TSC) (Scollay et al., 1988; Boyd and Hugo, 1991). Although histologically the thymus appears to be structurally simple, the complexity of T-cell maturation in this organ would predict that the stromal cells are equally complex. Examination of cultured thymic stromal cells (Loor, 1979), ultrastructural studies (Van de Wijngaert et al., 1984), and distribution of MHC antigens (Van Ewijk et al., 1980) provided the first indications that this was so.

Recently, mAb to murine, human and rat TSC have demonstrated a limited number of specific regions in the thymus, in particular, the antigenically distinct nature of the cortical and medullary epithelium, Hassall's corpuscles, the subcapsular and medullary epithelium and neuroendocrine components (e.g., Haynes, 1984; van Vliet et al., 1984a: De Maagd et al., 1985; Lobach et al., 1985; von Gaudecker, 1986; Boyd et al., 1988; Colic et al., 1988; Godfrey et al., 1990; Izon and Boyd, 1990). Interspersed throughout these are macrophages, reticular fibroblasts, and dendritic cells (Haynes, 1984; van Vliet et al., 1984a; Crowley et el., 1989). Whether cells of the phagocytic thymic reticulum (Nabarra and Papiernik, 1988) are distinct from, or encompassed within, these cells is not known. We have previously confirmed and markedly extended these studies with a panel of extensively characterized mAb to mouse TSC (Boyd et al., 1988; Tucek et al., 1989; Godfrey et al., 1990). These were divis-

*Corresponding author.

ible into four major categories: extensive stellate networks, vascular-associated stromal cells, relatively rare isolated stromal cells apparently representing discrete microenvironments, and antigens restricted to thymic-dependent lymphoid tissues but expressed on both TSC and T lymphocytes.

In the present study, we have produced an extensive panel of mAb to the chicken thymus as a prelude to deciphering the microenvironment of this organ in this species. These reagents have demonstrated the highly conserved nature of thymus and have confirmed the complexity of its milieu. In addition, these mAb have also been used to monitor abnormalities in the thymus of autoimmunity-prone line 200 chickens (Boyd et al., 1991).

### RESULTS

### Classification

Where possible, the reactivities of the antithymic epithelium mAb have been classified according to the guidelines proposed at the 1989 Rolduc Thymic Epithelium Workshop. All the mAb listed have distinct reactivities even if they are included within the same group. As part of this workshop, 25 mAb to human, mouse, or rat thymic epithelium from different laboratories were extensively cross-characterized against reference thymuses, embryonic thymuses, and an extensive panel of nonthymic tissues. Based on concordant results from three or four different laboratories, and the original classifications proposed at the Oxford workshop (Ritter and Haynes, 1987), the mAb were subdivided into groups using CTES nomenclature (Clusters of Thymic Epithelial Staining patterns). The initial findings of this workshop have recently been published (Kampinga et al., 1989). The reactivity of the present panel of mAbs to the bursa, where relevant, has been described in detail elsewhere (Boyd et al., 1990).

### Thymic Reactivity mAb Reactive with Thymic Epithelium (Table 1).

Eleven mAb reacted only with epithelial cells within the thymus. MUI-73 was defined as a panepithelium reagent as it clearly stained the entire subcapsule, subtrabecular, and stellate networks in the cortex and medulla (Fig. 1a), completely mimicking the antikeratin reagent. It does not react with all epithelium on other tissues, for example, in the bursa, only the basement membrane-associated epithelium and weak surface epithelium were positive, whereas the extensive medullary reticular epithelial networks were negative (Boyd et al., 1990). According to the Rolduc classification, MUI-73 is in category CTES-1. MUI-54 stained all the epithelium of the medulla and cortex and the majority, but not all of the type-I epithelium in the subcapsule/subtrabecular and perivascular regions in the medulla. Hassalls' corpuscles are less obvious in the chicken than in man, but small epithelial cell clusters resembling these structures were positive (Figs. 1b and 1c). This antibody is not exclusively epithelium-specific, however, as it showed a fine, extracellular, granular pattern throughout the cortex and medulla.

Several mAb identified subsets of epithelial cells. MUI-70 and MUI-75 were specific for the type-I epithelium lining the subcapsular/subtrabecular and perivascular regions (Figs. 1d and 1e). Subspecificities within this type-I epithelium were revealed by MUI-51 and MUI-53, which, in the subcapsule, only stained restricted regions (Figs. 1f and 1g). MUI-52 identified isolated cortical stellate epithelial cells (Figs. 1h and 1i); they were less frequent although similar in morphology to MHC class-II (MUI-78) positive cells (see Fig. 2g).

MUI-58 demonstrated the antigenic similarity between the medullary and subcapsular thymic epithelium (Fig. 1j) (CTES II). This mAb had very restricted nonthymic reactivity, but of particular interest, it stained isolated stellate cells in splenic germinal centres (Fig. 1k) and the basement membrane-associated epithelium in the bursa (Boyd et al., 1990).

Three mAb reacted with different components of the thymus medullary epithelium. MUI-71 stained a stellate network of epithelial cells (CTES IV) that constituted approximately 70% of the medullary epithelium; in contrast to MUI-58, it was negative on the subcapsule. MUI-62 and MUI-69 reacted with distinct clusters of medullary epithelial cells. Both appeared to label the cytoplasm, but whereas MUI-69 staining was confined to the cells within the clusters, the

TABLE 1
Monoclonal Antibodies Reactive to Only Thymic Epithelial Cells

| mAb (MUI) | Isotype | Thymic localization | CTES classification[a] |
|---|---|---|---|
| 73, | IgM | Panepithelium | CTES I |
| 54 | IgM | Pancortex and medullary epithelium, minority subcapsule and perivascular | CTES XX.a |
| 70,75 | IgM,IgM | Subcapsule, perivascular epithelium (pan-type I) | Not defined |
| 51,53 | IgM,IgM | Subpopulation of type-I epithelium | Not defined |
| 52 | IgM | Cortex: isolated stellate epithelium | Not defined |
| 58 | IgM | Subcapsule, perivascular; Medulla: stellate network | CTES II |
| 71 | IgM | Medulla: stellate epithelial network | CTES IV |
| 62 | IgG3 | Medulla: epithelial clusters; granular | CTES $V_C$ |
| 69 | IgM | Medulla: epithelial clusters | CTES IVa |

[a]CTES classification as defined by Kampinga et al., 1989.

determinant detected by MUI-62 was more granular and extracellular, indicative of a secreted product (Figs. 1l and 1m). The MUI-62-positive cells may be the chicken equivalent of Hassalls' corpuscles (HC) and would be of category CTES V.C. MUI-69 only stained a subset of medullary epithelial cells that also appears to include HC.

A remarkable finding that was consistently observed in the double labeling experiments with antikeratin was the presence of extensive areas completely lacking epithelial cells. These keratin-negative areas (KNA) were predominantly in the central regions of the thymic lobes. They are not extrathymic regions as they contain numerous T lymphocytes (see over).

mAb Reactive with Thymic Epithelial and Non-Epithelial Stromal Cells (Table 2).

MUI-66 reacted with clusters of morphologically less differentiated epithelial cells in the thymic medulla and corticomedullary junction and isolated, stellate keratin-negative cells throughout the cortex, in perivascular spaces and the KNA (Figs. 2a and 2b). These latter cells resembled reticular fibroblasts (van Vliet et al., 1984a). MUI-72 had a similar thymic distribution to MUI-66 because it also stained the less stellate medullary epithelial clusters and reticular fibroblast-like cells in the KNA and perivascular spaces (Figs. 2c and 2d); interestingly, however, the cortex was negative. MUI-80 strongly stained medullary epithelial clusters and isolated frequent keratin-negative cells throughout the cortex, medulla, KNA, and perivascular spaces (Figs. 2e and 2f). Although similar to MUI-66, the keratin-negative cells were less fibroblastic in appearance.

MUI-78 stained frequent, isolated stellate epithelial cells in the thymic cortex and was confluent in the medulla (Figs. 2g and 2h). The medullary staining was primarily of keratin-negative cells, but also included keratin-positive cells. This distribution is consistent with that of MHC class-II (BL). Interestingly, isolated cells within the KNA were also stained by MUI-78.

mAb Reactive with Non-Epithelial Thymic Stromal Cells (Table 3).

MUI-56 clearly delineated the capsule and trabeculae—no other thymic structures were stained (Fig. 3a). This involved both the cells and fibers of this connective tissue component. MUI-79 identified isolated keratin-negative cells throughout the thymic cortex, medulla, and in the perivascular spaces (Figs. 3b and 3c). Morphologically, the cells resembled macrophages, being irregular in shape with cytoplasmic processes; they were not fibroblastic. MUI-82 stained only the endothelium of the major blood vessels in the medulla (Fig. 3d). Capillaries in the subcapsule, trabeculae, and cortex were negative.

### Distribution of T-Cell Markers in KNA

T cells present in the KNA were mainly CD4-positive and to a lesser extent CD8-positive (Figs. 4a and 4f). T cells expressing the $\gamma\delta$-TCR (TCR-1) were also frequent in the medulla and ECF, but only scattered in the cortex; $\alpha\beta$-TCR (TCR-2)

were present only as infrequent, isolated cells in the KNA (N. Davidson, personal communication).

### Reactivity on Thymic Stromal-Cell Suspensions

MUI-78 gave the most significant staining of thymic stromal-cell suspensions, again similar to MHC class II. It stained thymic nurse cells (TNC) and subpopulations of stromal cells morphologically, resembling both epithelial cells and macrophage-like cells. When the isolated stromal cells were separated by elutriation (Andrews and Shortman, 1985) and cultured *in vitro*, the first fractions (smallest stromal cells) were enriched for MUI-78$^+$ cells, which resembled dendritic cells. MUI-36, -51, -58, -62, -69, and -72 were plasma membrane reactive as they stained approximately 5%–10% of the fresh, viable stromal cells. Of particular interest, MUI-51, -58, and -75 reacted with the plasma membrane of intact TNC. There was no significant staining of T lymphocytes by any antibodies other than the T-cell markers CD3, CD4, CD8, and MUI-83, except for MUI-78, which reacted with most Con-A-activated blasts and approximately 20% of thymocytes.

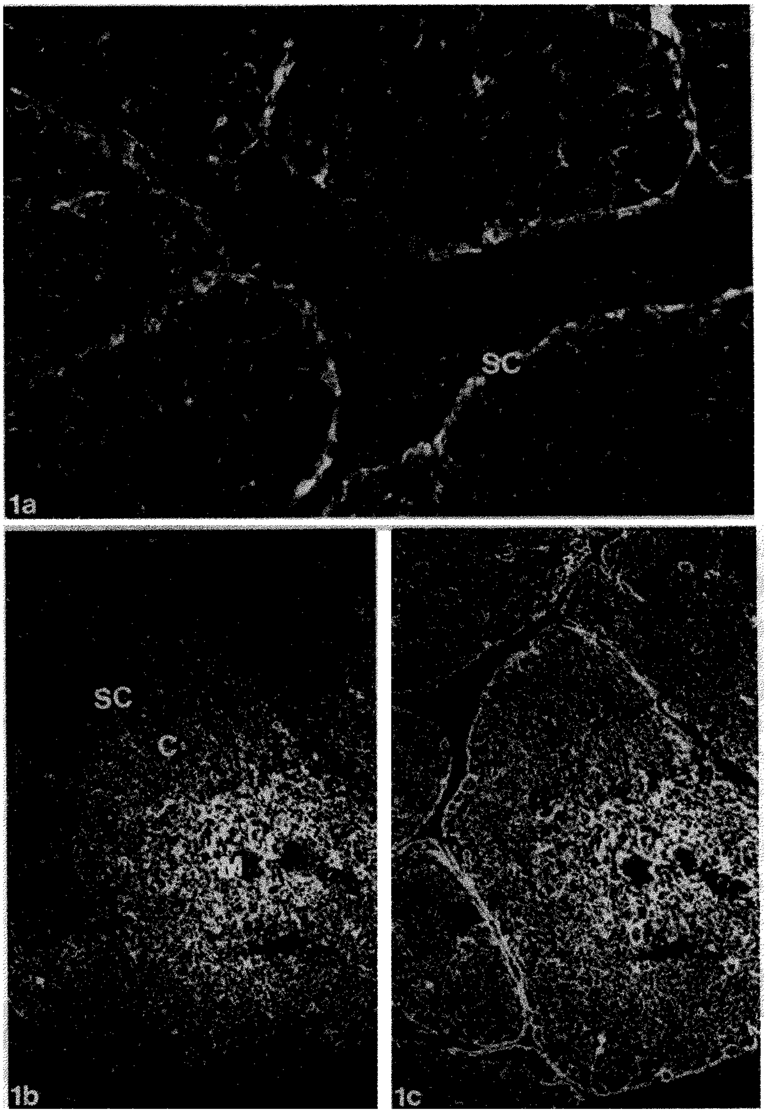

FIGURE 1. Immunofluorescence staining of mAb reactive with chicken thymic epithelial cells. (a) MUI-73 (panepithelium) staining of the subcapsule (SC) and epithelial network in the cortex (×280).(b) MUI-54 staining of cortical (C) and medullary (M) epithelium but only limited subcapsular epithelium (SC). (×125). (c) Double labeling of (b) with antikeratin. (×125).

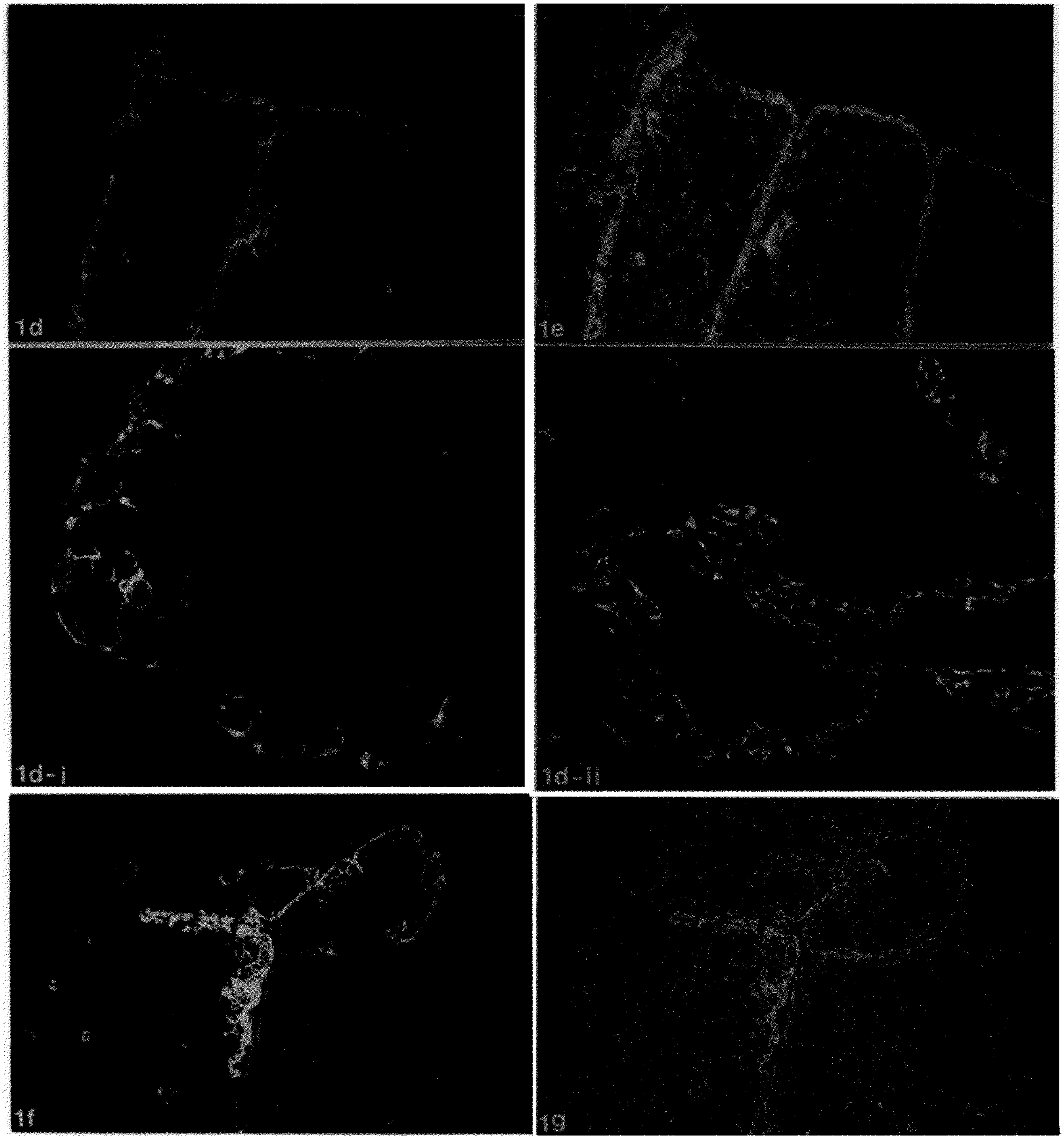

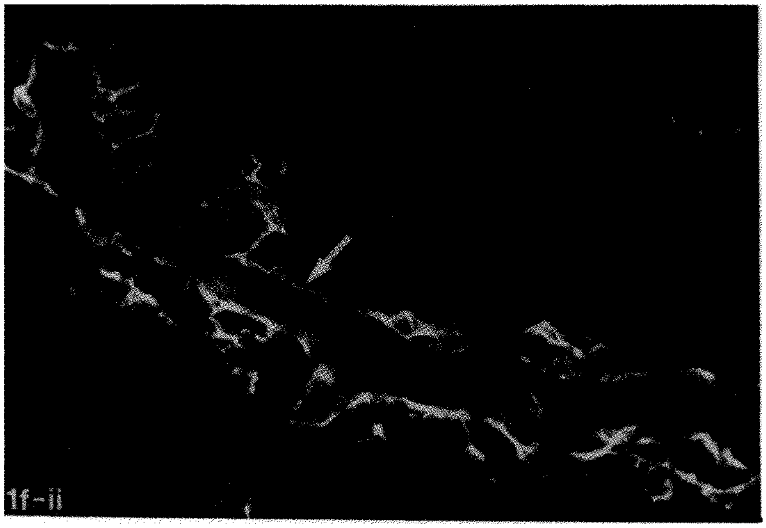

FIGURE 1. Immunofluorescence staining of mAb reactive with chicken thymic epithelial cells. (d) MUI-70 (pan-type-I epithelium) staining of subcapsular epithelium and its extensions down the trabeculae (×125). Further detail of reactivity with the subcapsular (d-i) and medullary perivascular (d-ii) regions (×280). (e) Double labeling of (d) with antikeratin (×125). (f) MUI-53 staining of restricted areas of type-I epithelium in the subcapsular/subtrabecular and medullary perivascular regions (× 125). Further detail (f-ii) of the subcapsular reactivity showing staining of both cells and the lamina propria (arrow) (×280). (g) Double labeling of (f) with antikeratin (×125).

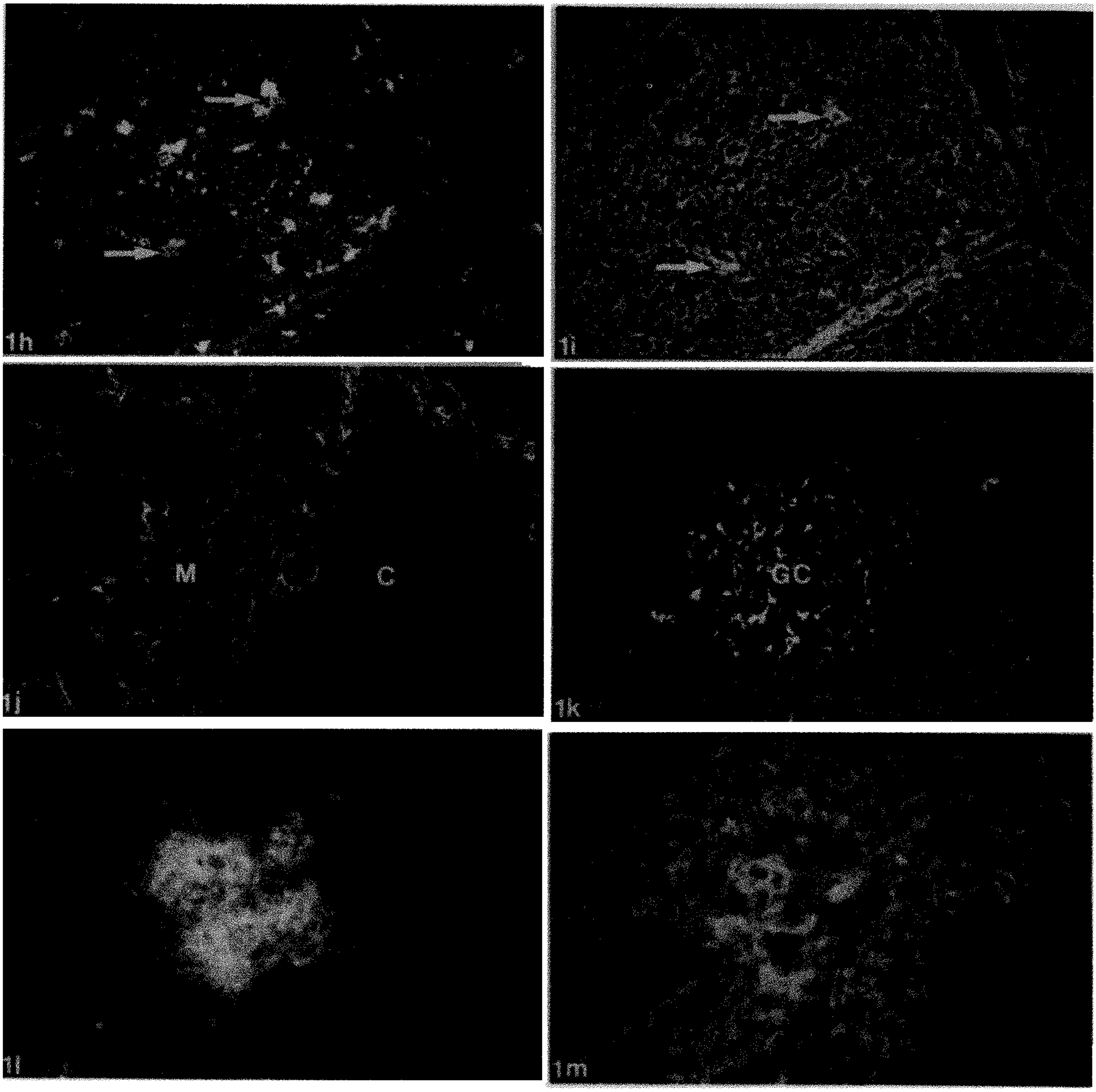

FIGURE 1. Immunofluorescence staining of mAb reactive with chicken thymic epithelial cells. (h) MUI-52 staining of isolated, stellate, keratin-positive cells (arrows) in the cortex (×125). (i) Double labeling of (h) with antikeratin (×125). (j) MUI-58 staining of stellate medullary (M) but not cortical (C) epithelial cells (×280). (k) MUI-58 staining of stellate stromal cells in a germinal centre (GC) in the spleen (×280). (l) MUI-62 staining of clusters of epithelial cells in the medulla. Note the granular extracellular fluorescence (×280). (m) Double labeling of (l) with antikeratin (×280).

### Nonlymphoid Reactivity

During the initial screening process, those hybridomas with broad tissue cross-reactivity were discarded. Despite this, many of the mAb showed reactivity, if only restricted, with non-thymic tissue, particularly the bursa (Boyd et al., 1990). The nonlymphoid reactivity of the mAb is summarized in Table 4.

mAb Reactive with Thymic Epithelium.

The panthymic epithelium mAb also stained many other tissues, although not all epithelium; for example, MUI-54 stained only the basal epithelial layer of the tongue and was negative on thyroid and lacrimal gland. The type-I epithelium mAb (MUI-51, -53, -70, and -75) stained restricted forms of epithelium in non-thymic tis-

TABLE 2
Monoclonal Antibodies Reactive to Both Thymic Epithelial and Non-Epithelial Stromal Cells

| mAb (MUI) | Isotype | Thymic localization[a] |
|---|---|---|
| 66 | IgG1 | Medulla: epithelial clusters; Cortex: PVS, KNA: isolated non-epithelial cells |
| 72 | IgM | Medulla: epithelial clusters; KNA: isolated stellate cells |
| 80 | IgG2b | Medulla: isolated stellate epithelial cells; Cortex: PVS, KNA: isolated non-epithelial cells |
| 78 | IgG2a | Medulla: confluent epithelial and non-epithelial cells; Cortex: isolated epithelial cells; (MHC class-II monomeric determinant) |

[a]PVS: perivascular space; KNA: keratin-negative areas

TABLE 3.
Monoclonal Antibodies Reactive to Only Thymic Non-Epithelial Stromal Cells

| mAb (MUI) | Isotype | Thymic localization |
|---|---|---|
| 56 | IgM | Connective tissue associated with capsule, trabeculae, and perivascular regions |
| 79 | IgM | Medulla, PVS: isolated stellate stromal cells |
| 82 | IgG1 | Medulla: vascular endothelium |
| 36 | IgG2a | Medulla: B cells, subset macrophages |
| 83 | IgG1 | Cortex: T cells confluent; Medulla: scattered T cells |

sues. In particular, MUI-75 identified ovarian follicular epithelium and vascular endothelium of the lung. MUI-53 demonstrated the similarities in the perivascular epithelium of many tissues. MUI-52, which stained isolated thymic cortical epithelial cells, labeled isolated cells in the tunica propria of the respiratory and gastrointestinal tracts, basal epithelium of the tongue, and the stratum germinatum, follicles, and dermis of the skin. It also stains developing epithelial subsets in the bursa (Wilson and Boyd, 1990) and most cultured thymic and bursal epithelial cells (unpublished observations).

MUI-62 (isolated medullary epithelial cells) identifies a mucin-like molecule present in the glandular epithelium throughout the intestinal and respiratory tracts (Bean et al., in preparation). MUI-69 had a similar thymic specificity, but only stained infrequent bursal follicles (Boyd et al., 1990) and infrequent mucosal glands.

mAb Reactive with Thymic Epithelial and Non-Epithelial Cells.

MUI-66 and -72 (stellate medullary epithelial clusters and isolated reticular fibroblasts) differed in their nonthymic specificities. MUI-66 had a broader epithelial reactivity than MUI-72 and stained reticular fibroblasts scattered throughout the splenic red pulp, gastointestinal tracts, and around hepatic sinusoids. The only non-epithelial reactivities of MUI-72 were of very infrequent isolated cells in the lamina propria of the gut. A striking feature of both these mAb was their strong reactivity with the bursal cortex (Boyd et al., 1990). MUI-80 stained the surface epithelium and glands of the gut and respiratory tract, the stratum germinatum and feather follicles of the skin, apical and basal epithelium of the tongue, lung bronchi, and Harder's gland. There was very little non-epithelial cell staining by MUI-80 in nonthymic tissues; in the spleen, MUI-80 showed strong granular staining only of reticular cells forming the periellipsoidal sheaths.

On nonlymphoid organs, MUI-78 reacted with isolated lymphocytic and stromal cells including Langerhans cells in the skin and brain astrocytes.

mAb Reactive with Non-Epithelial Thymic Stromal Cells.

The only staining of MUI-56 on non-thymic tissues was isolated fibroblastic-like cells between intestinal and heart muscle fibers, and connective tissue in the dermis, tonsil, around ovarian follicles, and isolated perivascular fibers in the spleen and liver. Consequently, although reacting with connective tissue (fibroblasts), the tissue distribution was restricted, many organs (e.g., lung, brain, kidney, and adrenals) being negative. MUI-79 stained a subset of macrophages in the thymus. When analyzed by flow cytometry, MUI-79 also stained 60–70% of blood monocytes and isolated macrophage-like cells in virtually all tissues tested, including the splenic red pulp, the interfollicular regions of the bursa, alveolar macrophages, Kupffer cells, glial cells, and in the laminar propria throughout the respiratory and gastrointestinal tracts. On non-thymic tissues, MUI-82 showed extensive staining of fibres in the splenic red pulp and restricted

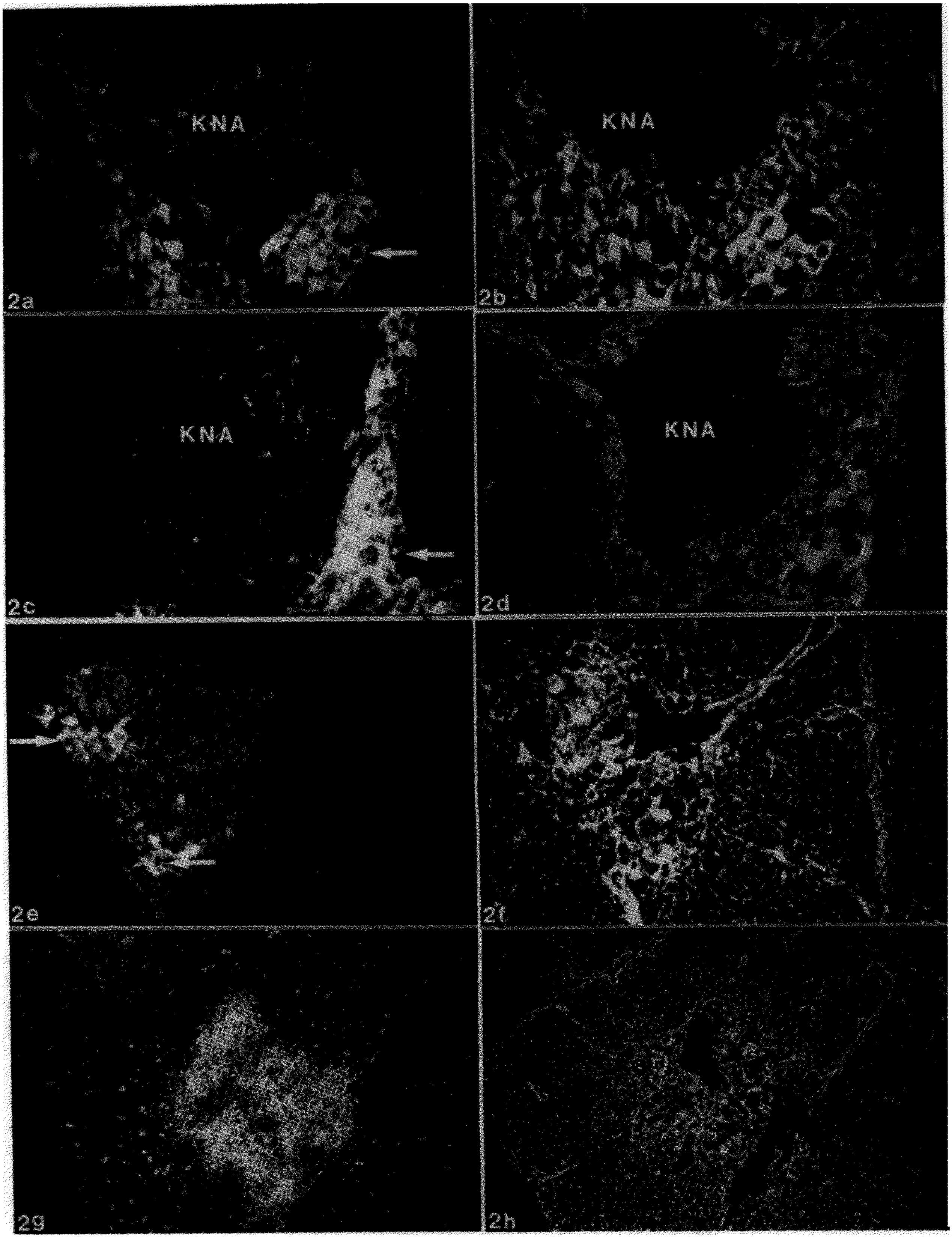

FIGURE 2. Immunofluorescence staining of mAb reactive with chicken thymic epithelial and non-epithelial stromal cells. (a) MUI-66 staining of clusters of morphologically less differentiated medullary epithelial cells (arrow) and isolated stellate stromal cells in the keratin-negative areas (KNA) (×250). (b) Double labeling of (a) with antikeratin (×250). (c) MUI-72 staining of medullary epithelial clusters (arrow) and isolated stellate cells in a KNA encompassing a perivascular space. Note the negative cortex (×250). (d) Double labeling of (c) with antikeratin (×250). (e) MUI-80 staining of medullary epithelial clusters (arrows) and isolated stellate keratin-negative cells predominantly throughout the medulla (×250). (f) Double labeling of (e) with antikeratin (× 250). (g) MUI-78 staining of isolated, stellate stromal cells in the cortex and confluent, predominantly epithelial, cells in the medulla. This pattern is typical of MHC class-II distribution (×125). (h) Double labeling of (g) with antikeratin (×125).

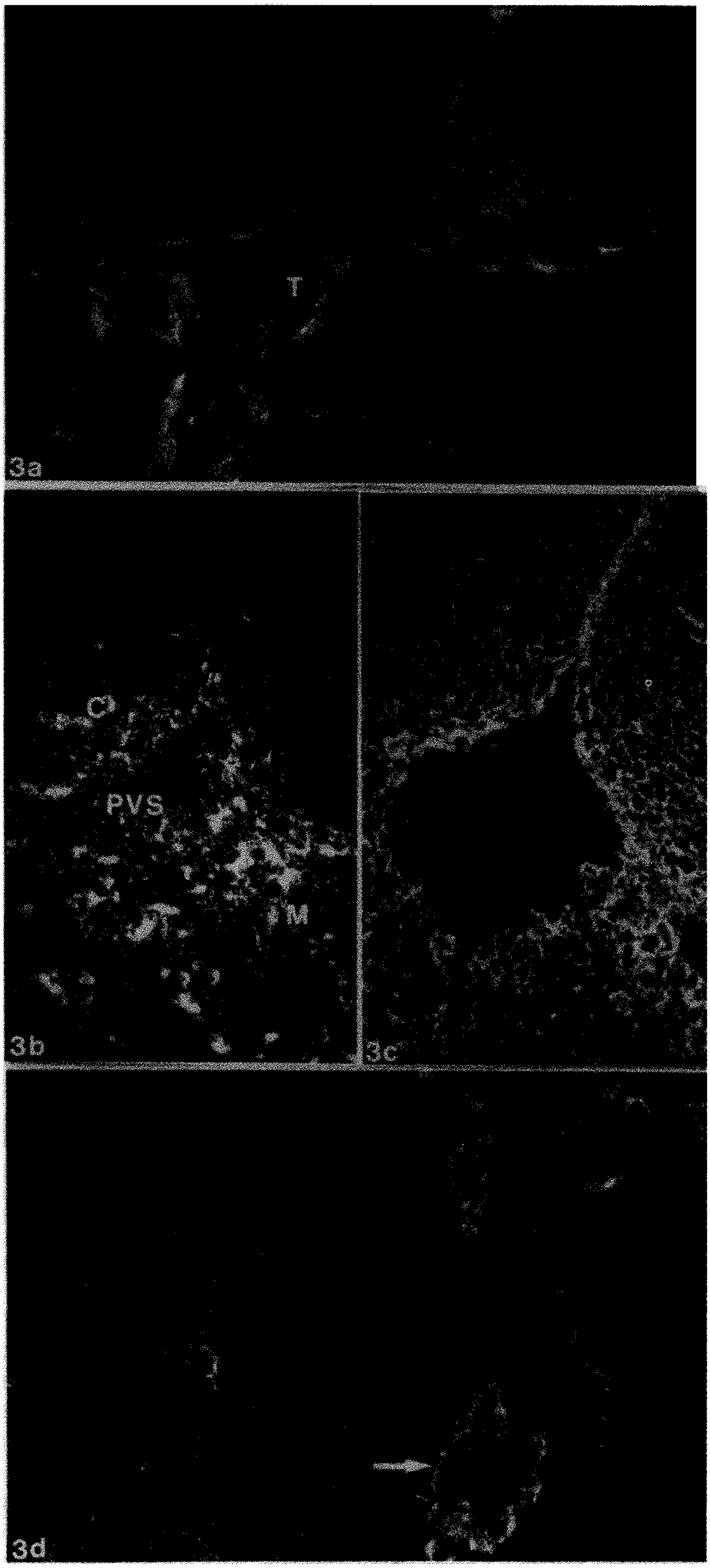

FIGURE 3. Immunofluorescence staining of mAb reactive with chicken thymic nonepithelial stromal cells. (a) MUI-56 staining of the thymic trabeculae (T). The capsule was also positive, but the entire medulla and cortex were negative (× 280). (b) MUI-79 staining of isolated macrophage-like cells in the medulla (M), perivascular space (PVS), and, to a lesser degree, the cortex (C) (×250). (c) Double labeling of (b) with antikeratin (×250). (d) MUI-82 staining of medullary vascular endothelium (arrow). The capillary network throughout the cortex, subcapsule, and trabeculae was negative (×280).

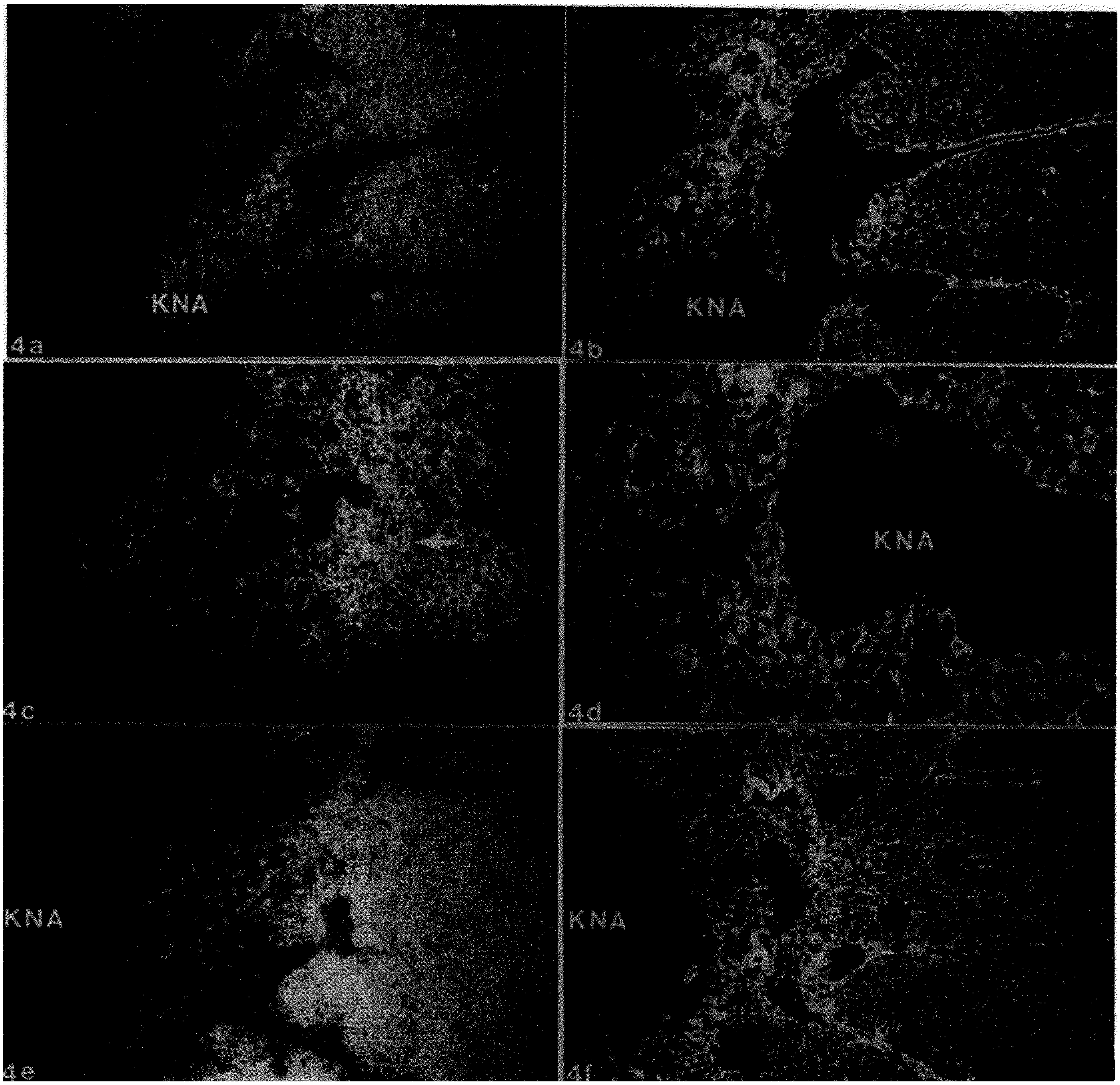

FIGURE 4. Intrathymic localization of CD4 and CD8. (a) Confluent staining of anti-CD4 throughout the cortex and isolated positive cells in the medulla. Note the extensive staining in the keratin-negative areas (KNA). (×125). (b) Double labeling of (a) with antikeratin, clearly demonstrating large central areas that lack epithelial cells (KNA) (×125). (c) Higher magnification of a KNA (arrow) showing extensive staining with anti-CD4 (×250). (d) Double labeling of (c) with antikeratin (×250). (e) Confluent staining of anti-CD8 throughout the cortex and isolated positive cells in the medulla. Note the less frequent positive cells in the KNA in comparison with anti-CD4 (Fig. 4a) (×125). (f) Double labeling of (e) with antikeratin (×125).

vasculature in the pancreas, parathyroid, kidney, and respiratory tract.

### DISCUSSION

In recent years, there has been increasing awareness of the importance of the stromal components of the thymic microenvironment in the induction and regulation T-cell differentiation. It is generally accepted that the major stromal populations resolve into those derived from bone marrow migrants, mesenchymal connective tissue, or epithelium. Each of these populations has different embryological origins (von Guadecker, 1986). This diversity was substantiated by analy-

TABLE 4.
Nonlymphoid Reactivity of Monoclonal Antibodies[a]

| mAb (MUI) | Epithelial cell labeling of mAb | Non-epithelial cell labeling of mAb |
|---|---|---|
| Group A: | mAb reactive to thymic epithelial cells | |
| 51 | HG;IT;Liv;Lu;Ov;Ton | Kid |
| 52 | HG;Ov;PT;Sk;Ton | Ad;IT;Kid;Liv;Lu;Ov;RT;Sk |
| 53 | IT;Ov;RT;Ton | BM in numerous tissues; Lu;M:Thy:Ton |
| 54 | Ad;HG;IT;Kid;Li;Lu;Ov; PT;RT;Sk;Ton | Br;Kid;Liv;LG;Lu;M;Ov;Thy |
| 58 | Ov | Ad;Thy |
| 62 | IT;Kid;Ov;P;RT;Sk;Ton | Ov |
| 69 | IT | —[b] |
| 70 | IT;Ov | Br;HG;Sk;Ton |
| 75 | Ov | Liv;Lu;Ov |
| Group B: mAb reactive to both thymic epithelial and non-epithelial stromal cells | | |
| 66 | HG;IT;Kid;Lu;Ov;PT;RT;Sk;Ton | Ad;HG;IT;Ov;RT;Thy |
| 72 | Ad;HG;IT;Kid;Li;Ov;Ton | IT;Ov |
| 78 | RT | Isolated cells in numerous tissues, especially RT |
| 80 | HG;IT;Kid;Li;Lu;Ov; PT;RT;Sk;Ton | Ad;Ov |
| Group C: mAb reactive to thymic non-epithelial stromal cells | | |
| 36 | — | Isolated cells in numerous tissues (mainly B cells) |
| 56 | — | Isolated cells in IT;M;Ov;RT;Sk |
| 79 | — | Isolated cells in numerous tissues (macrophages) |
| 82 | — | Blood vessels in Kid;M;P;PT;RT |
| 83 | — | Isolated cells (subpopulation of T cells) |

[a]Ad: adrenals; BM: basement membrane; Br: brain; HG: Harder's gland; IT: intestinal tract; Kid: kidney; LG: lacrimal gland; Liv: liver; Lu: lung; M: muscle; Ov: ovaries; P: pancreas; PT: parathyroid; RT: respiratory tract; Sk: skin; Thy: thyroid; Ton: tongue.
[b]No significant staining.

sis of the distribution of MHC molecules, which demonstrated that MHC class I was primarily expressed on medullary stromal cells but only isolated cortical cells (van Ewijk et al., 1980), including the outer cortical located TNC (Wekerle et al., 1980; Boyd et al., 1984; van Vliet et al., 1984b). In contrast, MHC class-II molecules are expressed on the cortical epithelial network and confluent in the medulla (e.g., van Ewijk et al., 1980). Recently, a novel MHC class-II epitope was localized to a subset of medullary stromal cells (Murphy et al., 1989), indicating that T cells have a different exposure to MHC molecules than other cell types. The present study clearly demonstrates that the chicken thymic stroma is equally complex, and, in addition, markedly extends the mammalian data through the demonstration of new determinants.

Although the mAbs should prove invaluable for the functional dissection of the thymic microenvironment and phenotypic mapping of the stroma in various developmental and pathological conditions, an important question that remains to be resolved is the nature of the molecules that they identify. This is also true of the vast majority of mAbs to mammalian thymic stromal cells (Brekelmans and van Ewijk, 1990), the problem being that many of the molecules they detect are in relatively low abundance and can be difficult to isolate. As a consequence, the biochemical characterization of thymic stromal molecules is the major current focus of an international workshop (Rolduc CTES nomenclature) that is being conducted in several laboratories, on the analysis of anti-mammalian thymic epithelium reactive mAbs. The phenotypic classification of these workshop mAbs has recently been presented (Kampinga et al., 1989; Ladyman et al., 1991). In terms of the current mAbs to the chicken thymus, MUI-78 was confirmed as detecting a monomorphic MHC class-II determinant by molecular weight analysis. Under nonreducing conditions, there were two bands of Mr 28,000 and Mr 33,000; these correspond to the reported values for the BL-$\beta$ and $\alpha$ chains, respectively (Ewert et al., 1984; Guillemot et al., 1984). We have also determined that MUI-62 is a glycoprotein of two molecular forms of Mr 200K and 60K and MUI-36 is a homodimer of Mr 90K (Bean et al., in preparation).

The first group of mAb in the present study detected several subsets of chicken thymic epithelium. Collectively, this is an important finding because of the current interest regarding the influence of thymic epithelial cells on positive and negative selection during intrathymic T-cell differentiation. Thus, it is likely that it is not the simple expression of MHC antigens per se that regulate T- cell maturation, but their combination with specific stromal-cell subset molecules. MUI-73 was a panthymic epithelium reagent reacting with the type-I epithelium of the subcapsule and perivascular spaces and the entire cortical and medullary epithelium. Its thymic reactivity resembles that of IP, AE1, and HTS-1 in humans, R-MC3 in rats, and MTS 1, 5, and 39 in the mouse (Lobach et al., 1985; Boyd et al., 1988; Colic et al., 1988; Godfrey et al., 1990; Izon and Boyd, 1990). It is not a pankeratin because it failed to react with epithelium from all tissues and its reactivity was not blocked in the double-labeling experiments with antikeratin. MUI-73 would be classified as CTES-1 according to the Rolduc Workshop recommendations (Kampinga et al., 1989). MUI-54 also stained most of the thymic epithelium, but only reacted with a minority of the subcapsule and perivascular regions. It thus resembles the rat thymic marker HIS 46 and is of the CTES.xx.a category (Kampinga et al., 1989). Again, it is not an antikeratin because of the additional very fine, although weak, granular staining observed throughout several organs, including the thymus, bursa, brain, spleen, and heart. Such reactivity demonstrates that epithelial and non-epithelial cells share determinants that may therefore serve a "housekeeping" function.

MUI-51, -53, and -70 are particularly interesting because of their specificity for the flattened epithelium lining the subcapsule, subtrabeculae (which is an extension of the subcapsule), and the perivascular space. This has been defined as type-I epithelium because of its distinctive location adjacent to the basal lamina and characteristic morphology (van de Wijngaert et al., 1984); as yet, however, there have been no specific antigenic markers to precisely identify these cells in the thymus. Although the thymic MUI-70-positive epithelial cells are a distinct subset, they cannot be assigned a distinct state of epithelial-cell maturation because basal epithelial cells were positive in tongue, which appear to be the most immature cell types (Cotsarelis et al., 1989). However, in the gut, the apical epithelial cells of the villi were positive and these are the most mature cells (McClintic, 1978). The function of the type-I epithelial cells is unknown, but it can be proposed that since the subcapsule is the chief zone of thymic lymphopoiesis (Mandel, 1969), it is the epithelial cells that provide the mitotic stimulus. MUI-51 and -53, by their restricted staining patterns, further revealed specialized areas within the type-I epithelium. It may be related to different states of maturation within the type-I epithelium because MUI-51 stained the basal epithelium in the tongue and Harder's glands and the intestinal crypts—all regions of less differentiated epithelium (Cotsarelis et al., 1989).

The antigenic similarity between the subcapsular and medullary epithelium was revealed by MUI-58. This has also been described in humans, mice, and rats (Haynes, 1984; De Maagd et al., 1985; Colic et al., 1988; Kampinga et al., 1989; Godfrey et al., 1990; Izon and Boyd, 1990), demonstrating a remarkable phylogenetically conserved relationship between the subcapsule and medulla; it is indicative of their proposed common endodermal origin in the embryo (Haynes, 1984). MUI-58 was negative on all nonthymic tissues with the exception of pancreatic tubules, some ovarian follicles, and the oviduct mucosal epithelium. This very restricted reactivity would be consistent with a functional role for the epitope in T-cell differentiation.

Three mAb were specific for thymic medullary epithelium. MUI-62 labeled small clusters of epithelial cells, which may include the chicken equivalent of Hassall's corpuscles. The granular extracellular staining indicates either secretion of a soluble product of the central epithelial cells or concentration within these cells of a factor produced by adjacent cells. The reactivity pattern including its presence in intestinal goblet cells and bursal medulla, closely resembles a gut-associated mucin (CGAMA) previously described (Boyd and Ward, 1984; Houssaint et al., 1986). MUI-69 has a similar intrathymic distribution to MUI-62, but was not obviously extracellular. It was also more restricted in its nonthymic reactivity; in particular, it stained only some of the bursal follicles (Boyd et al., 1990). MUI-69, therefore, may be a good candidate as having a specific role in T- and B-cell differentiation, and reflects similarities between primary lymphoid organs.

MUI-71 reacted with approximately 70% of the medullary epithelium, these cells forming a stellate network. Medullary epithelial cells can thus be distinguished by sets of markers: pan-medulla (MUI-58); subset network (MUI-71); isolated small clusters, perhaps including Hassall's corpuscles (MUI-62, -69); and pockets of epithelial cells (MUI-66, -72). The thymic epithelial subset detected by MUI-66 has relevance to the concept of a stromal-cell precursor (Lampert and Ritter, 1988) because the cells are morphologically undifferentiated and MUI-66 stained the stratum germinatum in the skin and crypts of the gut. The last two areas consist of pluripotential epithelial stem cells that generate the overlying areas of more differentiated epithelium (Cotsarelis et al., 1989). Whether the MUI-66-positive cells are precursors to the others and hence the medullary subsets would be all distinct phases of the one lineage is uncertain, but has been recently addressed (Wilson et al., in press).

In addition to the epithelial cell heterogeneity revealed by the MUI mAb, a remarkable finding to emerge was the extensive areas of the thymus that lack epithelium (keratin-negative areas; KNA), particularly in the central, medullary regions. Van Ewijk et al. (1980) alluded to similar regions in the mouse thymus, based on the distribution of MHC class-I and -II antigens. Guillemot et al. (1984) also indicated that large areas of MHC class-II positive non-epithelial cells were present in the chicken thymus. We initially interpreted these as being extrathymic, but staining with anti-CD3, CD4, CD8, and TCR-1 clearly demonstrated the presence of T cells and hence that they were an integral part of the thymic parenchyma. The functional significance of the KNA is still unknown, but in view of the importance of epithelial cells in positive selection through their expression of plasma membrane MHC molecules, they may be areas of accumulation of T cells undergoing, or which have undergone, negative selection, thought to be mediated by bone-marrow-derived cells, in particular, dendritic cells (Owen et al., 1986). MUI-66, -72, -78, and -80 all stained isolated non-epithelial cells in the KNA. In this regard, the staining pattern of MUI-78 in both thymic and nonthymic tissues was identical to that ascribed to B-L, the chicken MHC class-II product (Boyd et al., 1984; Ewert et al., 1984; Guillemot et al., 1984; Hala et al., 1984; Wick et al., 1984). MUI-72 (and MUI-78) may be detecting dendritic cells (Owen et al., 1986) because it was negative in the cortex and in chimeric studies, the vast majority of MHC class-II-positive cells in the chicken thymus medulla are bone-marrow-derived (Guillemot et al., 1984). In mammals, dendritic cells are also concentrated at the corticomedullary junction/medullary areas (van Ewijk, 1988). MUI-72 also stained isolated, stellate non-epithelial cells in nonlymphoid tissues and in the bursal cortex, but the precise identity of these is not yet resolved. Regardless, MUI-72 is not specific for dendritic cells because it also stains a subset of thymic medullary epithelium, although in mammals, thymic dendritic cells share common epitopes with cortical epithelium (Schuurman et al., 1987; Colic et al., 1988). It will be important to purify the two medullary subsets identified by MUI-72, because one could be involved in negative selection (e.g., dendritic cells) and the other (epithelial cells) could provide the final differentiation stimulus for maturing T cells. The isolated stellate cells throughout the thymus detected by MUI-66 and MUI-80 may be the chicken equivalent of murine reticular fibroblasts. MUI-79 and MUI-36 react with macrophages: whereas the latter detected only a very minor subpopulation, the former appears to be a lineage marker for monocytes and macrophages, similar to CHNL-68-1 (Jeurissen et al., 1988).

Collectively, these mAb represent excellent tools for the study of thymic stromal cell control of chicken T-cell differentiation, in particular, for determining the nature of cells secreting chemoattractants (Dunon et al., 1990) and those responsible for tolerance induction (Salaun et al., 1990). As a first approach to this, we have examined the ontogenic development of the stromal determinants relative to T cells (Wilson et al., in press) and intrathymic abnormalities associated with the autoimmunity-prone UCD line 200 chickens (Van de Water et al., 1990; Boyd et al., 1991).

### MATERIALS AND METHODS

### Chickens

Closed colony Australorp×White Leghorn F1 hybrid chickens were used as the source of tissues for immunization and characterization of all the mAb. These were obtained from Research Poultry Farm (Research, Victoria) and maintained under standard animal house conditions at Monash University Medical School.

### Stromal-Cell Preparations

Suspensions of chicken thymic stromal cells were prepared as described (Boyd et al., 1984). Briefly, thymi were removed from freshly killed 6–8-week-old chickens and extensively teased at 4 °C in RPMI 1640 containing 0.25% collagenase and 0.01% DNAse. At 5-min intervals, the tissues were vigorously resuspended. After 20 min, the cells in the supernatant were collected and the remaining tissue digested to a single cell suspension in a fresh enzyme solution; this was usually complete within 20 to 30 min. Cell suspensions were pooled and then gently layered over inactivated newborn calf serum (NBS) (1 ml of suspension/3 ml NBS) and sedimented at unit gravity for 30 min, 4 °C, to fractionate the stromal cells. Cells on top of the interface and in the upper 0.5 ml were discarded as they were predominantly lymphocytes. The remaining cells were collected by centrifugation (250 g, 5 min) and consisted primarily of TNC, macrophages, epithelial cells, and vasculature. This stromal-cell-rich suspension was used for immunization and for cell suspension staining.

### Monoclonal Antibodies

Adult Balb/c mice (8–12 weeks old) were immunized intraperitoneally three times at weekly intervals, with approximately 100 $\mu$l packed stromal-cell-rich suspension, in 300 $\mu$l RPMI 1640. Spleens were removed 3 days after the final injection and fused with log-growth phase P3-NS-1-Ag-4 (NS-1) cells at a ratio of 1:1, and plated into 96-well flat-bottom tissue-culture plates. The hybridoma supernatants were initially screened by indirect immunofluorescence on 4-$\mu$m cryostat-cut sections of snap-frozen composite blocks of thymus, bursa, and spleen using an affinity-purified sheep antimouse immunoglobulin FITC conjugate (DDAF, dilution 1:100, Silenus, Melbourne, Australia). Selected hybridomas were cloned at least twice by limiting dilution. Control preparations were stained with NS-1-conditioned medium or an unrelated mAb. All sections were mounted in veronal buffered glycerol containing 1 mg/ml p-phenylenediamine to prevent fading and examined with a Zeiss Axioskop microscope. Photography was performed using a Zeiss MC100 camera and Kodak Ektachrome P800/1600 film.

Additonal mAb reactive with chicken CD3, CD4, CD8, and TCR-1($\gamma\delta$-like) were kindly provided by Drs. C.-L. Chen and M.D. Cooper (Chen et al., 1986; Chan et al., 1988; Sowder et al., 1988).

### Specificity

All mAb studied herein were initially tested on cryostat sections of thymus, bursa, spleen, colon, crop, heart, distal and proximal duodenum, ileum, kidney, caecal tonsil, lung, esophagus, gizzard, skin, brain, trachea, pancreas, tongue, cloaca, pharynx, ovary, liver, gall bladder, adrenals, oviduct nerve, and Harder's gland. Reactivity with epithelial cells was determined by double labeling with a rabbit antikeratin (broad spectrum; dilution 1:200; DAKO, Santa Barbara, CA) developed with rhodamine conjugated goat-anti-rabbit immunoglobulin (dilution 1:50, Silenus). In addition, flow cytometry analyses (gating on 0 ° and 90 ° scatter profiles for lymphoid, stromal, and total cell populations) were performed on stromal-cell suspensions of the thymus and standard cell suspensions of thymus, bursa, spleen, bone marrow, and blood leukocytes using a FACScan (Becton Dickinson). Con-A-activated spleen cells were also FACScan analyzed.

### Isotyping of mAb

The isotype of the mAb was determined by agar gel immunodiffusion using culture supernatants concentrated ten-fold by negative pressure dialysis and subclass specific reagents (Nordic Immunology, Tilburg, The Netherlands).

*(Received April 3, 1991)*

*(Accepted July 19, 1991)*

### REFERENCES

Andrews P., and Shortman K. (1985). Zonal unit-gravity elutriation. A new technique for separating large cells and multicellular complexes from cell suspensions. Cell. Biophysics **7:** 251–266.

Boyd R.L., and Hugo P. (1991). Towards an integrated view of thymopoiesis. Immunol. Today **12:** 71–79.

Boyd R.L., Izon D.J., Godfrey D.I., Wilson T.J., Ward H.A., and Tucek C.L. (1988). Complex heterogeneity of the thymic stroma. Adv. Exp. Med. Biol. **237:** 263–268.

Boyd R.L., Oberhuber G., Hala I.K., and Wick G. (1984). Obese strain (OS) chickens with spontaneous autoimmune thyroiditis have a deficiency in thymic nurse cells. J. Immunol. **132:** 718–724.

Boyd R.L., and Ward H.A. (1984). Lymphoid antigenic determinants of the chicken: Ontogeny of bursa-dependent lymphoid tissue. Dev. Comp. Immunol. **8:** 148–167.

Boyd R.L., Wilson T.J., van de Water J., Haapanen L.A., Ward H.A., and Gershwin M.E. (1991). Selective abnormalities in the thymic microenvironmental abnormalities associated with avian scleroderma, an inherited fibrotic disease of line 200 chickens. J. Autoimmunity. **4:** 369–380.

Boyd R.L., Wilson T.J., Ward H.A., and Mitrangas K. (1990). Phenotypic characterization of chicken bursal stromal elements. Dev. Immunol. **1:** 41–51.

Brekelmans P., and van Ewijk W. (1990). Phenotypic characterization of murine thymic microenvironments. Semin. Immunol. **2:** 13–24.

Chan M.M., Chen C.H., Ager L.L., and Cooper M.D. (1988). Identification of the avian homologues of mammalian CD4 and CD8 antigens. J. Immunol. **140:** 2133–2138.

Chen C.H., Ager L.L., Gartland G.L., and Cooper M.D. (1986). Identification of a T3/T cell receptor complex in chickens. J. Exp. Med. **164:** 375–380.

Colic M., Matanovic L., Hegedis L., and Dujic A. (1988). Immunohistochemical characterization of rat thymic nonlymphoid cells. I. Epithelial and mesenchymal components defined by monoclonal antibodies. Immunology **65:** 277–284.

Cotsarelis G., Cheng S.-Z., Dong G., Sun T.-T., and Lavke R.M. (1989). Existence of slow-cycling limbal epithelial based cells that can be preferentially stimulated to proliferative implications on epithelial stem cells. Cell **57:** 201–209.

Crowley M., Inaba K., Witmer-Pack M., and Steinman R.M. (1989). The cell surface of mouse dendritic cells: FACS analyses of dendritic cells from different tissues including thymus. Cell. Immunol. **118:** 108–125.

De Maagd R.A., Mackenzie W.A., Schuurman H.-J., Ritter M.A., Price K.M., Broekhuizen R., and Kater L. (1985). The human thymus microenvironment: Heterogeneity detected by monoclonal anti-epithelial cell antibodies. Immunology **54:** 745–754.

Dunon D., Kaufman J., Salomonsen J., Skoedt K., Vaino O., Thiery J.P., and Imhof B.A. (1990). T cell precursor migration towards $\beta$2-microglobulin is involved in thymus colonization of chicken embryos. EMBO **9:** 3315-3322.

Ewert D.L., Munchus M.S., Chen C.-L.H., and Cooper M.D. (1984). Analysis of structural properties and cellular distribution of avian Ia antigen by using monoclonal antibody to monomorphic determinants. J. Immunol. **132:** 2524–2530.

Godfrey D.I., Izon D.J., Tucek C.L., Wilson T.J., and Boyd R.L. (1990). The phenotypic heterogeneity of mouse thymic stromal cells. Immunology **70:** 66–74.

Guillemot F.P., Oliver P.D., Peault B.M., and Le Douarin N.M. (1984). Cells expressing Ia antigens in the avian thymus. J. Exp. Med. **160:** 1803–1819.

Hala K., Wick G., Boyd R.L., Wolf H., Bock G., and Ewert D.L. (1984). The B-L (Ia-like) antigens of the chicken, lymphocyte plasma membrane distribution and tissue localization. Dev. Comp. Immunol. **8:** 673–682.

Haynes B.F. (1984). The human thymic microenvironment. Adv. Immunol. **36:** 87–142.

Houssaint E., Diez E., and Hallet M.-M. (1986). The bursal microenvironment: Phenotypic characterization of the epithelial component of the bursa of Fabricius with the use of monoclonal antibodies. Immunology **58:** 43–49.

Izon D.J., and Boyd R.L. (1990). The cytoarchitecture of the human thymus detected by monoclonal antibodies. Human Immunol. **27:** 16–32.

Jeurissen S.H.M., Janse E.M., Koch G., and de Boer G.F. (1988). The monoclonal antibody CVI-CHNL-681 recognizes cells of the monocyte-macrophage lineage in chickens. Dev. Comp. Immunol. **12:** 855–864.

Kampinga J., Berges S., Boyd R., Brekelmans P., Colic M., van Ewijk W., Kendall M., Ladyman H., Nieuwenhuis P., Ritter M., Schuurman H.-J., and Tournefier A. (1989). Thymic epithelial antibodies: Immunohistological analysis and introduction of CTES nomenclature. Summary of the Rolduc Epithelium Workshop. Thymus **13:** 165–173.

Ladyman H., Boyd R.L., Brekelmans P., Colic M., van Ewijk W., von Gaudecker B., Kampinga J., Kendall M., Nieuwenhuis P., Schuurman H.-J., Tournefier A., and Ritter M. (1991). Monoclonal antiepithelial antibody workshop II. Flow cytometric analysis and biochemical analysis of thymic epithelial cell heterogeneity. Proceedings of the 10th International Conference on Lymphatic Tissues and Germinal Centres in Immune Reactions (New York: Marcel Dekker).

Lampert I.A., and Ritter M.A. (1988). The origin of the diverse epithelial cells of the thymus: is there a common stem cell? *In:* Thymus update 1. The microenvironment of the human thymus, Kendall M.D and Ritter M.A., Eds. (Chur: Harwood Academic Publishers), p. 5.

Lobach D.F., Scearce R.M., and Haynes B.F. (1985). The human thymic microenvironment. Phenotypic characterization of Hassall's bodies with the use of monoclonal antibodies. J. Immunol. **134:** 250–257.

Loor F. (1979). Mouse thymus reticulo-epithelial (RE) cells in vitro: Isolation, cultivation and preliminary characterization. Immunology **37:** 157–177.

McClintic J.R. (1978). Physiology of the human body, 2nd Edition (New York: John Wiley and Sons).

Mandel T. (1969). Epithelial cells and lymphopoiesis in the cortex of guinea-pig thymus. Aust. J. Exp. Biol. Med. Sci. **47:** 153–158.

Murphy D.B., Lo D., Rath S., Brinster R.L., Flavell R.A., Slanetz A., and Janeway C.A., Jr. (1989). A novel MHC class II epitope expressed on thymic medulla but not cortex. Nature **338:** 765–768.

Nabarra B., and Papiernik M. (1988). Phenotype of thymic stromal cells. An immunoelectron microscopic study with anti-Ia, anti-Mac-1 and anti-Mac-2 antibodies. Lab. Invest. **58:** 524–531.

Owen J.J., Jenkinson E.J., and Kingston R. (1986). Thymic stem cells: Their interaction with the thymic stroma and tolerance induction. Curr. Top. Microbiol. Immunol. **126:** 35–41.

Ritter M.A., and Haynes B.F. (1987). Summary of thymic epithelium workshop. *In:* Leukocyte Typing III. White cell differentiation antigens McMichael A.J., Ed., (Oxford: Oxford University Press), p. 747.

Salaun J., Bandeira A., Khazaal I., Calman F., Coltey M., Coutinho A., and Le Douarin N.M. (1990). Thymic epithelium tolerizes for histocompatibility antigens. Science **247:** 1471–1474.

Schuurman H.-J., Ritter M.A., Broekhuizen R., Ladyman H., and Larche M. (1987). The thymic epithelium panel of antibodies: Immunohistologic analysis of human tissues. *In:* Leukocyte Typing. III. White cell differentiation antigens, McMichael A.J., Ed., (Oxford: Oxford University Press), pp. 259–262.

Scollay R., Wilson A., D'Amico A., Kelly K., Egerton M., Pearse M., Wu L., and Shortman K. (1988). Developmental status and reconstitution potential of subpopulations of murine thymocytes. Immunol. Rev. **104:** 81–120.

Sowder J.T., Chen C.-L.H., Ager L.L., Chan M.M., and Cooper M.D. (1988). A large subpopulation of avian T cells express a homologue of the mammalian T $\gamma/\delta$ receptor. J. Exp. Med. **167:** 315–322.

Tucek C.L., Boyd R.L., and Hiai H. (1989). Antigens shared by thymic stromal cells and T lymphocytes are abnormally expressed in AKR thymuses. Thymus **14:** 95–107.

van de Water J., Wilson T.J., Haapanen L.A., Boyd R.L., Abplanalp H., and Gershwin M.E. (1990). Ontogeny of T cell development in avian Scleroderma. Clin. Immunol. Immunopathol. **56:** 169–184.

van de Wijngaert F.P., Kendall M.D., Schuurman H.-J., Rademakers L.P.H.M., and Kater L. (1984). Heterogeneity of epithelial cells in the human thymus. An ultrastructural study. Cell. Tissue. Res. **237:** 227–237.

van Ewijk W. (1988). Cell surface topography of thymic microenvironments. Lab. Invest. **59:** 579–590.

van Ewijk W., Rouse R.V., and Weissman I.L. (1980). Distribution of H-2 microenvironments in the mouse thymus. Immunoelectron microscopic identification of I-A and H-2k bearing cells. J. Histochem. Cytochem. **28:** 1084–1099.

van Vliet E., Melis M., and van Ewijk W. (1984a). Monoclonal antibodies to stromal cell types of the mouse thymus. Eur. J. Immunol. **14:** 524–529.

van Vliet E., Melis M., and van Ewijk W. (1984b). Immunohistology of thymic nurse cells. Cell. Immunol. **87:** 101–109.

von Gaudecker B. (1986). The development of the human thymus microenvironment. Curr. Top. Pathol. **75:** 1–41.

Wekerle H., Ketelsen U.-P., and Ernst M. (1980). Thymic nurse cells. Lymphoepithelial complexes in the murine thymus: Morphological and serological classification. J. Exp. Med. **151:** 925–944.

Wick G., Hala K., Wolf H., Boyd R.L., and Schauenstein K. (1984). Distribution and functional analysis of B-L/Ia positive cells in the chicken: Expression of B-L/Ia antigens on thyroid epithelial cells in spontaneous autoimmune thyroiditis. Molec. Immunol. **12:** 1259–1265.

Wilson T.J., and Boyd R.L. (1990). The ontogeny of chicken bursal stromal cells defined by monoclonal antibodies. Dev. Immunol. **1:** 31–39.

